# Expression of receptors for epidermal growth factor and insulin-like growth factor I by ZR-75-1 human breast cancer cell variants is inversely related: the effect of steroid hormones on insulin-like growth factor I receptor expression.

**DOI:** 10.1038/bjc.1996.84

**Published:** 1996-02

**Authors:** H. W. van den Berg, D. Claffie, M. Boylan, J. McKillen, M. Lynch, B. McKibben

**Affiliations:** Department of Therapeutics and Pharmacology, The Queen's University of Belfast, UK.

## Abstract

We have investigated the expression of insulin-like growth factor I receptors (IGFR) by the ZR-75-1 human breast cancer cell line and tamoxifen-resistant (ZR-75-9a1) and oestrogen-independent (ZR-PR-LT) variants. ZR-75-1 cells expressed 6633+/-953 receptors per cell,(K(d) 0.24+/-0.06 nM). IGFR expression was reduced in ZR-75-9a1 cells (1180+/-614 receptors per cell, K(d) 0.13+/-0.05) and increased in the ZR-PR-LT cell line (18 430+/-3210 receptors per cell, K(d) 0.24+/-17). A comparison of these data with previously published findings for epidermal growth factor receptor (EGFR) expression by these cell lines revealed that IGFR and EGFR expression are inversely related in the variant lines whereas ZR-75-1 cells express similar numbers of both receptors. Since the changes in IGFR expression observed are associated with changes in steroid hormone receptor status, we also investigated the effects of oestradiol, the synthetic progestin ORG 2058 and dexamethasone on IGFR expression. Oestradiol increased IGFR expression only in the ZR-75-1 cell line. Low concentrations of ORG 2058 increased IGFR levels in the two cell lines positive for progesterone receptor (ZR-75-1 and ZR-PR-LT). High concentrations of ORG 2058 increased IGFR expression in all cell lines, as did dexamethasone. These data suggest that EGFR and IGFR expression may be linked in breast cancer, and that EGFR/IGFR ratios in breast cancer may be a more sensitive prognostic indicator than EGFR expression alone. Regardless of basal IGFR expression by the cell studied, ORG 2058 increased IGFR expression, possibly via both the progesterone and glucocorticoid receptors.


British Journal of Cancer (1996) 73, 477-481
© 1996 Stockton Press All rights reserved 0007-0920/96 $12.00

### Expression of receptors for epidermal growth factor and insulin-like growth factor I by ZR-75-1 human breast cancer cell variants is inversely related: the effect of steroid hormones on insulin-like growth factor I receptor expression

HW van den Berg[1], D Claffie[1], M Boylan[1], J McKillen[1], M Lynch[1] and B McKibben[2]

*Departments of [1]Therapeutics and Pharmacology and [2]Medicine, The Queen's University of Belfast, The Whitla Medical Building, 97 Lisburn Road, Belfast BT9 7BL, UK.*

**Summary** We have investigated the expression of insulin-like growth factor I receptors (IGFR) by the ZR-75-1 human breast cancer cell line and tamoxifen-resistant (ZR-75-9a1) and oestrogen-independent (ZR-PR-LT) variants. ZR-75-1 cells expressed 6633±953 receptors per cell ($K_d$ 0.24±0.06 nM). IGFR expression was reduced in ZR-75-9a1 cells (1180±614 receptors per cell, $K_d$ 0.13±0.05) and increased in the ZR-PR-LT cell line (18 430±3210 receptors per cell, $K_d$ 0.24±17). A comparison of these data with previously published findings for epidermal growth factor receptor (EGFR) expression by these cell lines revealed that IGFR and EGFR expression are inversely related in the variant lines whereas ZR-75-1 cells express similar numbers of both receptors. Since the changes in IGFR expression observed are associated with changes in steroid hormone receptor status, we also investigated the effects of oestradiol, the synthetic progestin ORG 2058 and dexamethasone on IGFR expression. Oestradiol increased IGFR expression only in the ZR-75-1 cell line. Low concentrations of ORG 2058 increased IGFR levels in the two cell lines positive for progesterone receptor (ZR-75-1 and ZR-PR-LT). High concentrations of ORG 2058 increased IGFR expression in all cell lines, as did dexamethasone. These data suggest that EGFR and IGFR expression may be linked in breast cancer, and that EGFR/IGFR ratios in breast cancer may be a more sensitive prognostic indicator than EGFR expression alone. Regardless of basal IGFR expression by the cell lines studied, ORG 2058 increased IGFR expression, possibly via both the progesterone and glucocorticoid receptors.

**Keywords:** insulin-like growth factor receptor; breast cancer; epidermal growth factor receptor; steroid hormone

Epidermal growth factor (EGF) and insulin-like growth factor I (IGF-I) are potent mitogens in human breast cancer and both act via membrane-associated receptors with intrinsic tyrosine kinase activity. The influence of IGF-I in regulating breast cancer cell proliferation appears to be under steroid hormone control at a number of levels. Oestrogen has been reported to up-regulate IGF-I receptor (IGFR) expression, possibly sensitising tumour cells to the mitogenic effect of IGF-1 (Stewart *et al.*, 1990) and a positive relationship between oestrogen receptor (ER) and IGFR expression has been reported (Pekonen *et al.*, 1988). Conversely, progestins appear to down-regulate IGFR numbers (Papa *et al.*, 1991; Owens *et al.*, 1993). Tamoxifen has been reported to reduce circulating levels of IGF-I (Pollack *et al.*, 1992), and the pattern of expression of IGF binding proteins (IGFBPs), which can both attenuate and potentiate the actions of IGF-I, is influenced by both oestrogens and antioestrogens (Lonning, 1992; Owens *et al.*, 1993; Lahti *et al.*, 1994; Manni *et al.*, 1994). It is generally accepted that tumour expression of receptors for epidermal growth factor (EGFR) is a powerful prognostic indicator in human breast cancer, with high EGFR numbers being associated with low ER content and poor clinical prognosis (Harris, 1989). Recent evidence has suggested that the EGF and IGF receptor systems may influence one another at several levels. Administration of EGF or oestradiol to ovariectomised mice increases uterine IGF-I mRNA production (Hana and Murphy, 1994) suggesting that activation of the IGF receptor system may be a common down-stream event for both oestradiol- and EGF-induced cell proliferation. It has been reported that EGF can regulate IGFBP expression (Andreatta van Leyen *et al.*, 1994; Hembree *et al.*, 1994). The relationship between EGFR and IGFR expression is less clear. Since a positive ER status is associated with a positivity for IGFR (Pekonen *et al.*, 1988) and low EGFR levels (Harris, 1989), an inverse relationship between EGFR and IGFR expression might be anticipated. However such a relationship has not been clearly demonstrated to date (Pekonen *et al.*, 1988).

We have previously shown that acquired tamoxifen resistance accompanied by loss of detectable ERs and progesterone receptors (PGRs) in the ZR-75-1 human breast cancer cell line (Van den Berg *et al.*, 1989) is associated with an increase in EGFR expression (Long *et al.*, 1992), in agreement with clinical findings (Harris, 1989). Conversely, an oestrogen-independent variant of the same cell line, which constitutively expresses high numbers of PGRs (Van den Berg *et al.*, 1990), has a much reduced EGFR content (Long *et al.*, 1992). In this study we have characterised IGFR expression by these cell lines and report that IGFR expression is inversely related to EGFR expression. In light of an accompanying positive association between IGFR and PGR expression, we have also investigated the effects of progestins on IGFR expression in these cell lines.

Correspondence: HW van den Berg
Received 12 July 1995; revised 21 September 1995; accepted 12 October 1995

### Methods

### *Cell lines*

The ZR-75-1 human breast cancer cell line was obtained from Flow Laboratories, Irvine, UK. Cells were routinely maintained in RPMI-1640 medium supplemented with 5% fetal calf serum, 100 IU ml$^{-1}$ penicillin and 100 $\mu$g ml$^{-1}$ streptomycin. ZR-75-9a1 cells are a tamoxifen-resistant variant of ZR-75-1 (Van den Berg *et al.*, 1989) routinely maintained in RPMI-1640 medium in the presence of 8 $\mu$M tamoxifen. All experiments described were carried out on cells that had been grown in tamoxifen-free medium for at least 3 days.

ZR-PR-LT cells are oestrogen independent (Van den Berg *et al.*, 1990) and routinely maintained in medium lacking known oestrogenic activity, (RPMI-1640 medium lacking phenol red and supplemented with heat-treated and dextran-coated charcoal-stripped fetal calf serum). Steroid and EGF receptor status of the cell lines is routinely determined and the phenotypes originally reported (Van den Berg *et al.*, 1989, 1990; Long *et al.*, 1992) have proved to be stable.

*Radioiodination of IGF-I*

Receptor grade IGF-I (10 μg, Penisula Laboratories, St. Helens, UK) was iodinated using the iodogen method (Fraker and Speck, 1978) as previously described for EGF (Long *et al.*, 1992). The radioiodination mixture was fractionated by reverse-phase high-performance liquid chromatography (HPLC) using a Waters Associates gradient system (Milford, MA, USA) fitted with an analytical Vydac C4 column. The eluting gradient was trifluoroacetic acid (TFA)/water (0.05%/99.95% v/v) to TFA/water/acetonitrile (0.05%/29.95%/70.0% v/v) and the flow rate was 1 ml $min^{-1}$. Fractions (0.5 ml) were collected and 25 μl aliquots were taken for determination of radioactivity. Three major iodinated peak fractions were identified and the fractions covering these peaks were pooled, aliquoted and stored at −20°C. These peaks were assumed to represent monoiodination of IGF-I at each of the tyrosine residues present in the peptide. Of the three iodinated peaks separated by HPLC, two demonstrated similar specific binding to ZR-75-1 cells and variants. The third peak showed no specific binding and may contain IGF-I iodinated at $Tyr^{60}$, which has previously been shown to have low affinity for the IGF-I receptor (Schaffer *et al.*, 1993).

*[$^{125}$I]IGF-I binding to ZR-75-1 cells and variants*

IGFR expression by cells was determined using a whole cell binding assay as previously described (Long *et al.*, 1992). Cells ($2 \times 10^5$) were plated into 24-place multiwell dishes and allowed to attach for 24 h. [$^{125}$I]IGF-I binding was determined by replacing the medium with RPMI-1640 medium (0.5 ml) supplemented with 1% bovine serum albumin containing [$^{125}$I]IGF-I (0.01–0.33 nM) in the absence or presence of a 100-fold excess of non-labelled IGF-I to determine non-specific binding. Following a 1 h incubation at 4°C medium was removed and wells rinsed twice with ice-cold phosphate-buffered saline. Aliquots of 500 μl of 1M sodium hydroxide were added to each well and plates incubated for 1 h at 37°C to extract radioactivity.

Radioactivity was determined by scintillation counting, (LKB Wallac 1410 LSC). Maximum binding capacity ($B_{max}$) and affinity ($K_d$) were calculated after linearisation of specific binding data (Keightly and Cressie, 1980).

*Steroid hormone modulation of [$^{125}$I]IGF-I binding*

Cells ($5 \times 10^4$) were plated into 24-place multiwell dishes and allowed to attach for 24 h. Medium was then replaced with medium containing a range of concentrations of oestradiol, the synthetic progestin ORG 2058 (Amersham International) or dexamethasone (Sigma-Aldrich, Poole, Dorset, UK). Cells were incubated for 6 days and [$^{125}$I]IGF-I binding assessed as described above using a single concentration of [$^{125}$I]IGF-I (0.2 nM). Data is presented as [$^{125}$I]IGF-I binding as a percentage of control after correcting for changes in cell numbers in the treated groups. Oestradiol treatment ($10^{-8}$–$10^{-7}$ M) increased ZR-75-1 cell numbers by $<10\%$, decreased ZR-PR-LT cell numbers by 10–40% (Van den Berg *et al.*, 1990) and had no significant effect on ZR-75-9a1 cells (Van den Berg *et al.*, 1989). Dexamethasone and ORG 2058 reduced cell numbers in all cell lines by 5–40% (Van den Berg *et al.*, 1993).

*Statistical analysis*

All experiments were carried out in triplicate and data analysed by a one-way analysis of variance using the Student Newman–Keuls test.

### Results

Figure 1 shows the concentration-dependent binding of [$^{125}$I]IGF-I to ZR-75-1 cells. Non-specific binding was typically less than 10% and linearisation of binding data suggested a single class of specific binding site. Maximum binding capacity and ligand affinity for the receptor for the three cell lines studied is shown in Table I. The oestrogen independent ZR-PR-LT line expressed approximately three times the number of IGFR compared with the parent ZR-75-1 line. Conversely, IGFR numbers were greatly reduced in the tamoxifen-resistant ZR-75-9a1 line, there being a more than 15-fold difference between IGFR numbers in this cell line compared with ZR-PR-LT cells. These changes in IGFR expression were not accompanied by any significant change in ligand affinity for the receptor (Table I).

Figure 2 compares IGFR expression in the three cell lines with previously published data for EGFR expression (Long *et al.*, 1992). It can be seen that there is a clear inverse relationship between IGFR and EGFR expression in the variant cell lines ZR-PR-LT and ZR-75-9a1 while ZR-75-1 cells express similar numbers of both receptors.

**Figure 1** Total (●-●), non-specific (▲-▲) and specific binding (■-■) of [$^{125}$I]IGF-I to ZR-75-1 cells.

**Table I** IGF-I receptor expression by ZR-75-1 cells and variants

| | $B_{max}$ receptors per cell | $K_d$ (nM) |
|---|---|---|
| ZR-75-1 | 6633 ± 953 | 0.24 ± 0.06 |
| ZR-75-9a1 | 1180 ± 614 | 0.13 ± 0.05 |
| ZR-PR-LT | 18 430 ± 3210 | 0.24 ± 0.17 |

Results are means and s.e.m. of three determinations

**Figure 2** EGFR (□) and IGFR (■) expression by ZR-75-1 cells and variants.

Exposure of ZR-75-1 cells to oestradiol resulted in a dose-dependent increase in [$^{125}$I]IGF-I binding, (Figure 3). Oestradiol failed to increase [$^{125}$I]IGF-I binding in the tamoxifen-resistant ZR-75-9a1 cells, which lack oestrogen receptors (Van den Berg *et al.*, 1989). Oestradiol ($10^{-9}$ and $10^{-8}$ M) caused an approximate 25% decrease in [$^{125}$I]IGF-I binding in ZR-PR-LT cells whereas higher concentrations were without effect. Figure 4 shows the effect of the progestin ORG 2058 on [$^{125}$I]IGF-I binding by ZR-75-1 cells and variants. Physiological concentrations of ORG 2058 ($10^{-9}$ and $10^{-8}$ M) caused a 40–50% increase in [$^{125}$I]IGF-I binding in ZR-75-1 and ZR-PR-LT cells, but had no significant effect on [$^{125}$I]IGF-I binding by ZR-75-9a1 cells. A 30–50% increase in binding was observed in all cell lines at $10^{-7}$ M ORG 2058. At higher concentrations [$^{125}$I]IGF-I binding was markedly stimulated in the ZR-PR-LT line, whereas the effect was less marked in the ZR-75-9a1 line or the parent line. Since pharmacological concentrations of ORG 2058 may exert effects via the glucocorticoid receptor we also investigated the effects of dexamethasone on [$^{125}$I]IGF-I binding. Dexamethasone caused a dose-dependent increase in [$^{125}$I]IGF-I binding in all three cell lines, the increase being greatest in the ZR-PR-LT cell line (Figure 5).

**Figure 3** The effect of oestradiol on specific binding of [$^{125}$I]IGF-I to ZR-75-I (■-■), ZR-PR-LT (▲-▲) and ZR-75-9a1 (●-●) cells. Data are presented as means ± s.e.m. of three observations. *$P<0.05$, **$P<0.001$ *vs* control.

### Discussion

We have previously shown that acquisition of tamoxifen resistance as exemplified by the ZR-75-9a1 human breast cancer cell line is associated with an increase in the expression of EGFR compared with the parent ZR-75-1 line (Long *et al.*, 1992) and loss of ER and PGR (Van den Berg *et al.*, 1989). This finding was in accord with clinical observations that ER-negative/EGFR-positive human breast cancers have a poor prognosis and are resistant to tamoxifen treatment (Nicholson, 1988; Harris, 1989). In contrast, the oestrogen-independent ZR-PR-LT line has much reduced EGFR numbers accompanying elevated PGR expression (Van den Berg, 1990). In this study we have shown that these changes in EGFR expression associated with tamoxifen resistance and oestrogen independence respectively are paralleled by opposite changes in IGFR expression (Table I). While the parent cell line expresses similar numbers of EGFR and IGFR, in the variant lines, EGFR and IGFR expression is inversely related (Figure 2). These data suggest that EGFR and IGFR expression in ZR-75-1 cells are linked, with changes in the level of expression of one receptor being reflected by an opposite change in the expression of the other. There is evidence to support the concept of receptor cross-talk for EGFR and IGFR. Administration of EGF to ovariectomised mice increases uterine IGF-I mRNA production (Hana and Murphy, 1994) and it has been reported that EGF can regulate IGFBP-3 expression, thus sensitising cells to the effects of IGF-I. (Andreatta van Leyen *et al.*, 1994; Hembree *et al.*, 1994). It is possible that EGF sensitising of cells to IGF may occur more effectively in the face of high EGFR/low IGFR levels, while sensitisation would be less effective when EGFR numbers are low, and perhaps unnecessary when corresponding IGFR numbers are high.

Since there is good evidence that ER and EGFR expression is inversely related in breast cancer, (Nicholson, 1988; Harris, 1989) and there is a positive relationship between ER and IGFR expression (Pekonen *et al.*, 1988; Railo *et al.*, 1994), it might be expected that an inverse relationship between IGFR and EGFR expression would exist. Clinical studies have failed to establish such a relationship (Pekonen *et al.*, 1988; Foekens *et al.*, 1989). The reasons for this are unknown, but may be due to inappropriate cut-off points for receptor positivity, occupation of receptors by endogeneous ligands and other factors. To our knowledge, our data are the first to show an inverse relationship between IGFR and EGFR expression in human breast cancer cell lines *in vitro*. Whereas EGFR expression is elevated only 3-fold in the ZR-75-9a1 tamoxifen-resistant line

**Figure 4** The effect of ORG 2058 on specific binding of [$^{125}$I]IGF-I to ZR-75-I (■-■), ZR-PR-LT (▲-▲) and ZR-75-9a1 (●-●) cells. Data are presented as means ± s.e.m. of three observations. *$P<0.05$, **$P<0.001$ *vs* control.

**Figure 5** The effect of dexamethasone on specific binding of [$^{125}$I]IGF-I to ZR-75-I (■-■), ZR-PR-LT (▲-▲) and ZR-75-9a1 (●-●) cells. Data are presented as means ± s.e.m. of three observations. *$P<0.05$, **$P<0.001$ *vs* control.

compared with the parent line (Figure 2), the EGFR/IGFR ratios in the two cell lines are 0.75 and 12.5 respectively. Should such a relationship exist *in vivo* it is possible that an EGFR/IGFR ratio may provide a more sensitive prognostic indicator for antioestrogen resistance than EGFR expression alone.

We have shown that oestradiol increases IGF binding by the oestrogen-sensitive ZR-75-1 cell line, (Figure 3), in agreement with earlier studies (Stewart *et al.*, 1990). As expected, oestradiol was without effect in the ZR-75-9a1 line, which lacks oestrogen receptors (Van den Berg *et al.*, 1989). Although the mechanism is unknown, the observation that low concentrations of oestradiol reduces [$^{125}$I]IGF-I binding by ZR-PR-LT cells would be consistent with our earlier findings that oestradiol inhibits ZR-PR-LT cell proliferation (Van den Berg *et al.*, 1990). Elevated expression of IGFR by the oestrogen-independent ZR-PR-LT line is consistent with the observation that another oestradiol-induced protein (PGR) is also overexpressed in this cell line in the absence of oestrogenic stimulation (Van den Berg *et al.*, 1990).

[$^{125}$I]IGF-I binding is also increased in all cell lines by the synthetic progestin ORG 2058, (Figure 4). A flat dose–response curve was noted for the parent line, with all concentrations causing a 40–50% increase in binding. Physiological concentrations of ORG 2058 ($10^{-9}$ and $10^{-8}$ M) increased [$^{125}$I]IGF-I binding only in the cell lines positive for the presence of PGR, (ZR-75-1 and ZR-PR-LT). Higher concentrations of ORG 2058 increased [$^{125}$I]IGF-I binding in all three cell lines, with the effects being most marked in the variant lines, where $10^{-5}$ M ORG 2058 approximately doubled [$^{125}$I]IGF-I binding. In these cell lines dexamethasone treatment causes a similar increase in binding (Figure 5), suggesting that a pharmacological concentration of ORG 2058 may be acting via the glucocorticoid receptor. Taken together, these data suggest that [$^{125}$I]IGF-I binding is increased in the parent line by ORG 2058 acting primarily via PGR. In the ZR-PR-LT line, both PGR and the glucocorticoid receptor seem to be involved, whereas in the ZR-75-9a1 line the increase in [$^{125}$I]IGF-I binding seems to be primarily glucocorticoid receptor mediated. Confirmation of the relative contributions of PGR and the glucocorticoid receptor in mediating the effects of ORG 2058 described will require the use of specific progestin and glucocorticoid antagonists.

The observation that low concentrations of the progestin ORG 2058 increase [$^{125}$I]IGF-I binding in the ZR-75-1 cell line is in contrast to a previous study that showed that [$^{125}$I]IGF-I binding by a breast cancer cell line is reduced by progestins (Papa *et al.*, 1991). However, a different cell line (T47D) and different progestins (progesterone and R5020) were used in the latter study and the down regulation of IGFR reported was attributed to a progestin-induced increase in IGF-II secretion. The failure of the present study to demonstrate IGFR down regulation may in part be explained by the report that ZR-75-1 cells do not secrete IGF-II (Osborne *et al.*, 1989). Taken together with these earlier studies, our findings emphasise the complexity of potential interactions between steroid and peptide growth factor receptors. To our knowledge, this report is the first to indicate that IGFR expression may also be increased by glucocorticoids, and that high concentrations of a progestin may increase [$^{125}$I]IGF-I binding via the glucocorticoid receptor. The physiological significance of these findings is unclear, as progestins and glucocorticoids are generally growth inhibitory towards breast cancer cells *in vitro* and down regulation of a receptor for a potent mitogen such as IGF-I would be more consistent with these anti-proliferative effects. In this context it is of interest that progestins and glucocorticoids have also been shown to increase EGFR expression in a number of human breast cancer cell lines (Ewing *et al.*, 1989).

In conclusion, we have demonstrated an inverse relationship between EGFR and IGFR receptor expression by human breast cancer cells *in vitro*. Regardless of basal IGFR expression by the cell lines studied, [$^{125}$I]IGF-I binding is increased following exposure to a progestin and this effect may be mediated via both PGRs and glucocorticoid receptors.

**Acknowledgements**
The support of the Department of Health and Social Services (NI) and the Medical Research Council (JM) is gratefully acknowledged.

### References

ANDREATTA VAN LEYEN S, HEMBREE JR AND ECKERT RL. (1994). Regulation of insulin-like growth factor 1 binding protein 3 levels by epidermal growth factor and retinoic acid in cervical epithelial cells. *J. Cell Physiol.*, **160**, 265–274.

EWING TM, MURPHY LJ, NG ML, PANG GYN. LEE CSL, WATTS CKW AND SUTHERLAND RL. (1989). Regulation of epidermal growth factor receptor by progestins and glucocorticoids in human breast cancer cell lines. *Int. J. Cancer*, **44**, 744–752.

FOEKENS JA, PORTENGEN H, VAN PUTTEN WLJ, TRAPMAN AMAC, REUBI JC, ALEXIEVA-FIGUSCH J AND KLIJN JGM. (1989). Prognostic value of receptors for insulin-like growth factor-I, somatostatin, and epidermal growth factor in human breast cancer. *Cancer Res.*, **49**, 7002–7009.

FRAKER PJ AND SPECK JC. (1978). Protein and cell membrane iodinations with a sparingly soluble chloramine, 1,3,4,6-tetrachloro-3a, 6a-diphenylglycoluril. *Biochem. Biophys. Res. Commun.*, **80**, 849–857.

HANA V AND MURPHY LJ. (1994). Interdependence of epidermal growth factor and insulin-like growth factor-I expression in the mouse uterus. *Endocrinology*, **135**, 107–112.

HARRIS AL. (1989). Epidermal growth factor receptors in breast cancer: Association with early relapse and death, poor response to hormones and interactions with Neu. *J. Biochem.*, **34**, 123–131.

HEMBREE JR, AGARWAL C AND ECKERT RL. (1994). Epidermal growth factor suppresses insulin-like growth factor binding protein 3 levels in human papillomavirus type 16-immortalized cervical epithelial cells and thereby potentiates the effects of insulin-like growth factor 1. *Cancer Res.*, **54**, 3160–3166.

KEIGHTLY DD AND CRESSIE NAC. (1980). The Woolf plot is more reliable than the Scatchard plot in analysing data from hormone receptor assays. *J. Steroid Biochem.*, **13**, 1317.

LAHTI EI, KNIP M AND LAATIKAINEN TJ. (1994). Plasma insulin-like growth factor I and its binding proteins 1 and 3 in postmenopausal patients with breast cancer receiving long term tamoxifen. *Cancer*, **74**, 618–624.

LONG B, McKIBBEN B, LYNCH M AND VAN DEN BERG HW. (1992). Changes in epidermal growth factor receptor expression and response to ligand associated with acquired tamoxifen resistance or oestrogen independence in the ZR-75-1 human breast cancer cell line. *Br. J. Cancer*, **65**, 865–869.

LONNING PE. (1992). Influence of tamoxifen on plasma levels of insulin-like growth factor-I and insulin-like growth factor binding protein-I in breast cancer patients. *Cancer Res.*, **52**, 4719–4723.

MANNI A, BADGER B, WEI L, ZAENGLEIN A, GROVE R, KHIN S, HEITJAN D, SHIMASAKI S AND LING N. (1994). Hormonal regulation of insulin-like growth factor II and insulin-like growth factor binding protein expression by breast cancer cells in vivo: evidence for stromal epithelial interactions. *Cancer Res.*, **54**, 2934–2942.

NICHOLSON S. (1988). Quantitative assays of epidermal growth factor receptor in human breast cancer: cut-off points of clinical relevance. *Int. J. Cancer*, **42**, 36–41.

OSBORNE CK, CORONADO EB, KITTEN LJ, ARTEAGA CI, FUQUA SAW, RAMASHARMA K, MARSHALL M AND LIT CH. (1989). Insulin-like growth factor-II (IGF-II) – a potential autocrine paracrine growth factor for human breast cancer acting via the IGF-I receptor. *Mol. Endocrinol.*, **3**, 1701–1709.

OWENS PC, GILL PG, DE YOUNG MA, WEGER MA, KNOWLES SE AND MOYSE KJ. (1993). Estrogen and progesterone regulate secretion of insulin-like growth factor binding proteins by human breast cancer. *Biochem. Biophys. Res. Commun.*, **193**, 467–473.

PAPA V, HARTMANN KKP, ROSENTHAL SM, MADDUX BA, SIITERI PK AND GOLDFINE ID. (1991). Progestins induce down-regulation of insulin-like growth, factor-I (IGF-I) receptors in human breast cancer cells – potential autocrine role of IGF-II. *Mol. Endocrinol.*, **5**, 709–717.

PEKONEN F, PARANEN S, MAKINEN T AND RUTANEN EM. (1988). Receptors for epidermal growth factor and insulin-like growth factor-I and their relation to steroid receptors in human breast cancer. *Cancer Res.*, **48**, 1343–1347.

POLLACK MN, HUYNH HT AND LEFEBVRE SP. (1992). Tamoxifen reduces serum insulin-like growth factor I (IGF-I). *Breast Cancer Res. Treat.*, **22**, 91–100.

RAILO MJ, SMITTEN KV AND PEKONEN F. (1994). The prognostic value of insulin-like growth factor-I in breast cancer patients. Results of a follow-up on 126 patients. *Eur. J. Cancer*, **30A**, 307–311.

SCHAFFER L, LARSEN UD, LINDE S, HEJNAES KR AND SKRIVER L. (1993). Characterisation of the three 125-I-iodination isomers of human insulin-like growth factor I (IGF1). *Biochim. Biophys. Acta*, **1203**, 205–209.

STEWART AJ, JOHNSON MD, MAY FEB AND WESTLEY BR. (1990). Role of insulin-like growth factor receptors in the estrogen stimulated proliferation of human breast cancer cells. *J. Biol. Chem.*, **265**, 21172–21178.

VAN DEN BERG HW, LYNCH M, MARTIN JHJ, NELSON J, DICKSON GR AND CROCKARD AD. (1989). Characterisation of a tamoxifen resistant variant of the ZR-75-1 human breast cancer cell line, ZR-75-9a1 and stability of the resistant phenotype. *Br. J. Cancer*, **59**, 522–526.

VAN DEN BERG HW, MARTIN JHJ AND LYNCH M. (1990). High progesterone receptor concentration in a variant of the ZR-75-1 human breast cancer cell line adapted to growth in oestrogen free conditions. *Br. J. Cancer*, **61**, 504–507.

VAN DEN BERG HW, LYNCH M AND MARTIN JHJ. (1993). The relationship between affinity of progestins and antiprogestins for the progesterone receptor in breast cancer cells (ZR-PR-LT) and the ability to down-regulate the receptor: evidence for hetero-specific receptor modulation via the glucocorticoid receptor. *Eur. J. Cancer*, **29A**, 1771–1775.


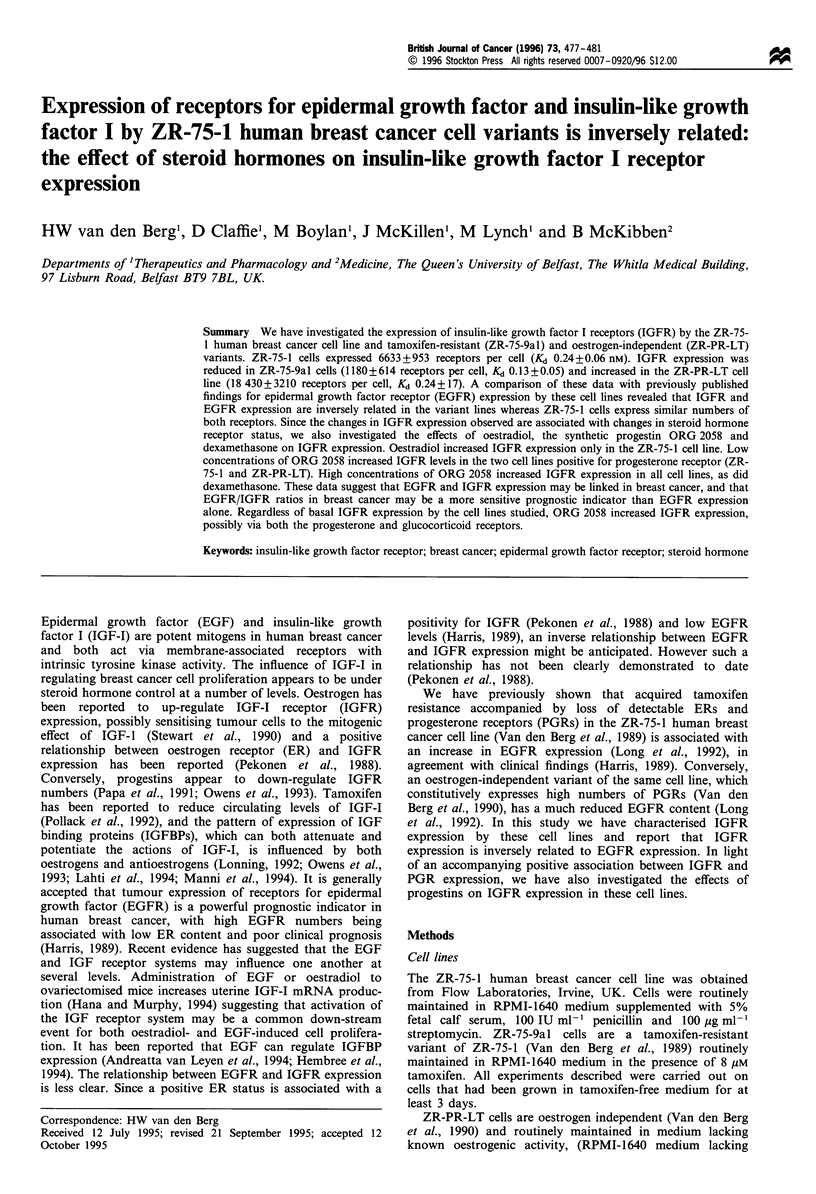

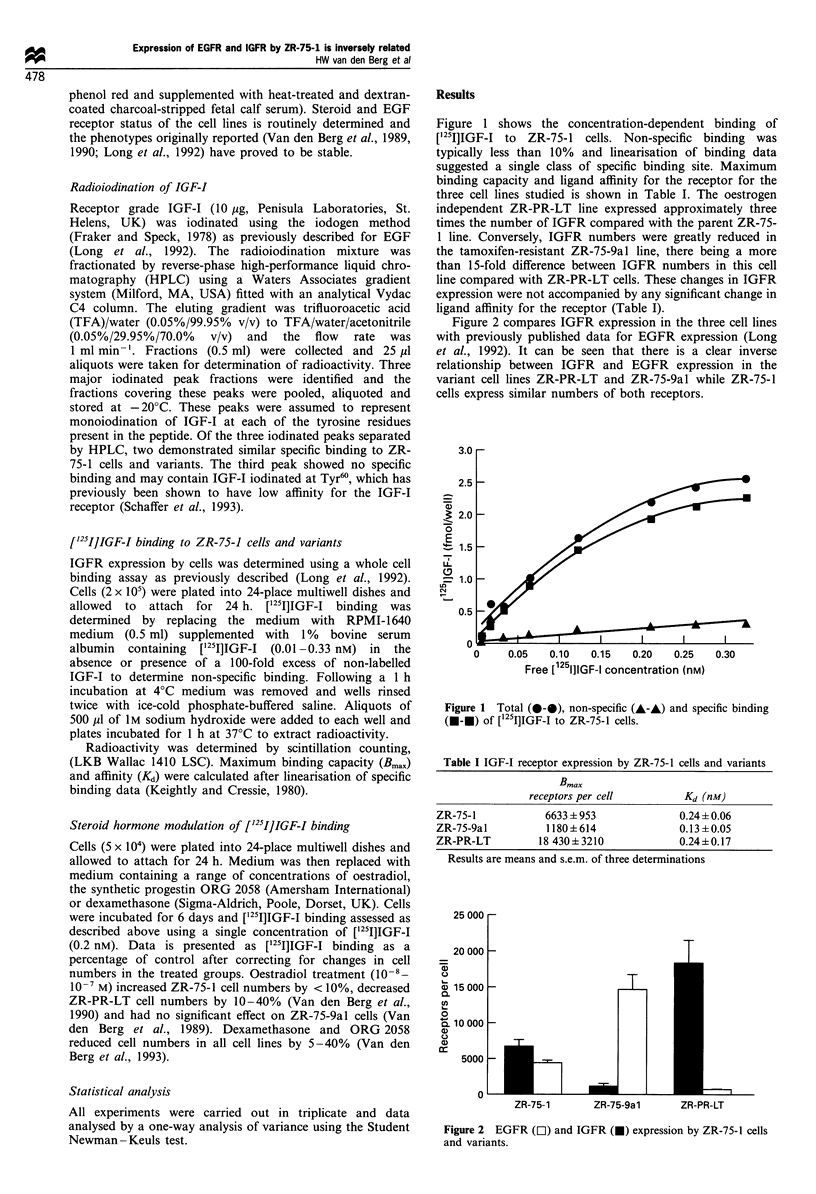

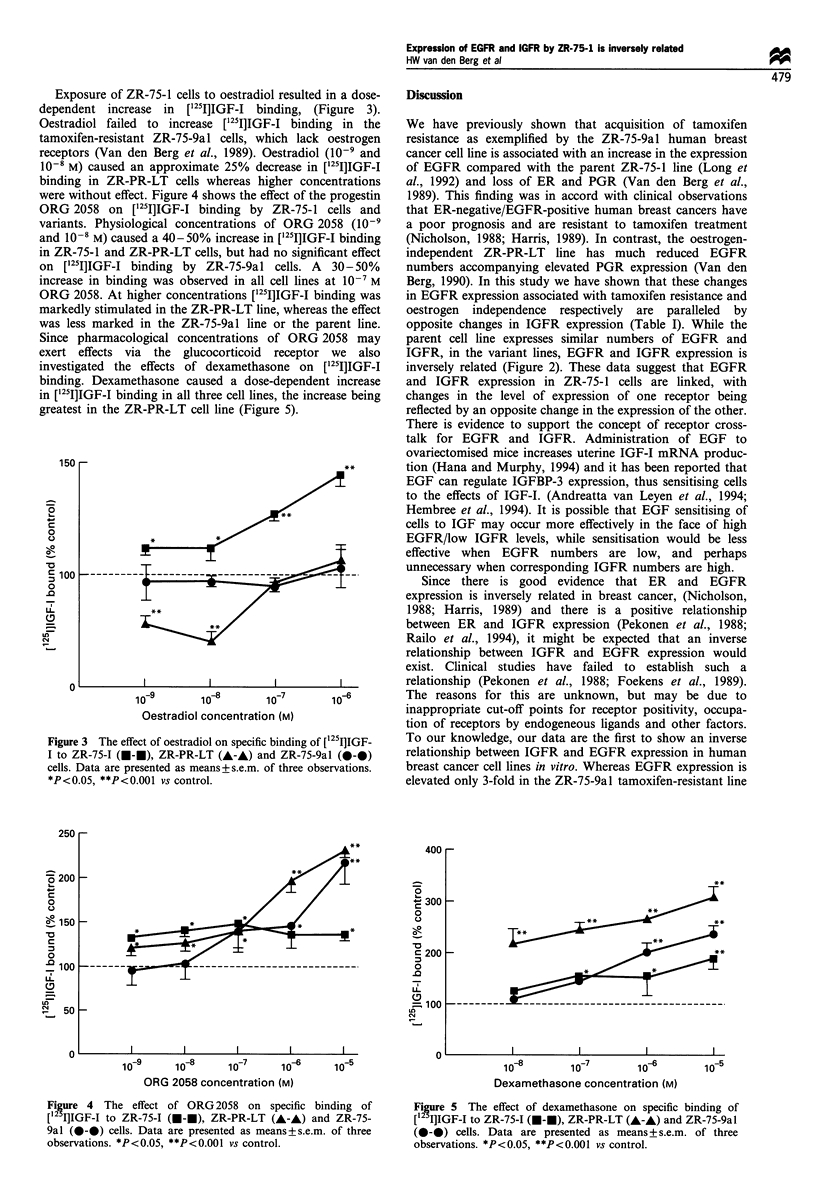

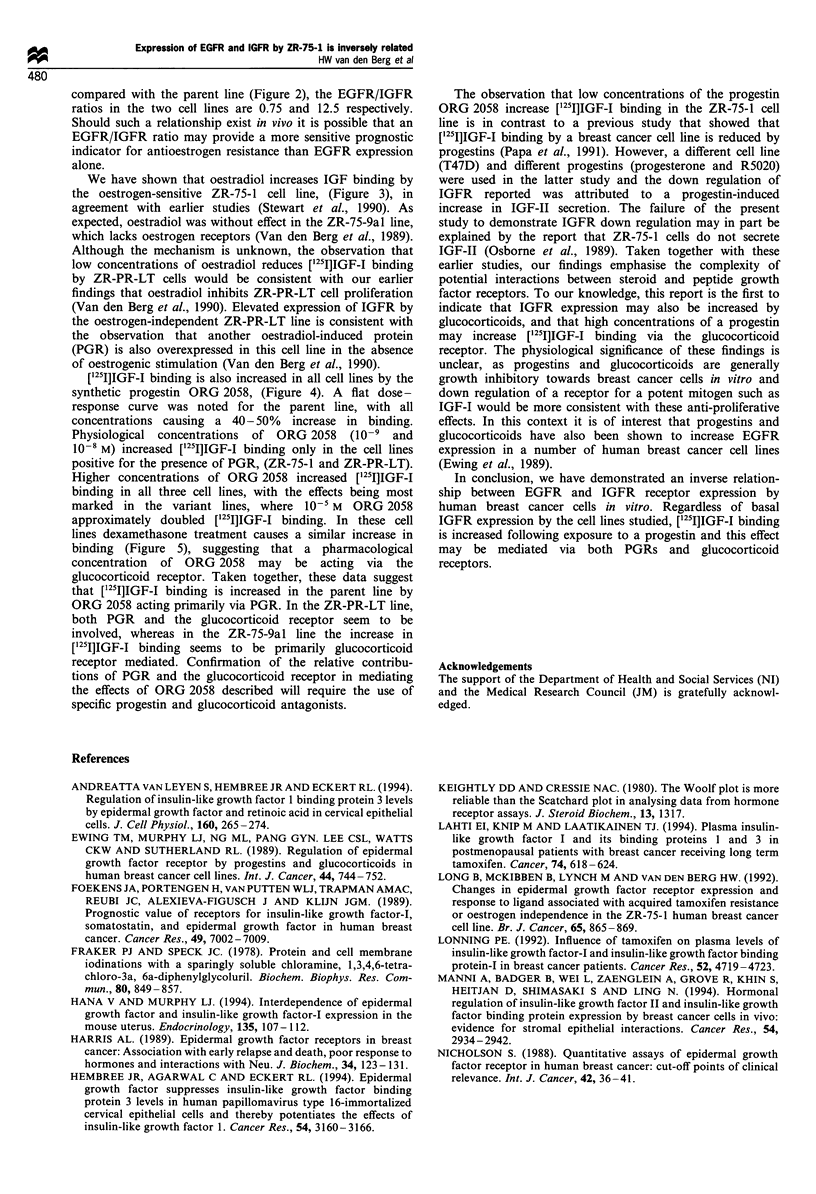

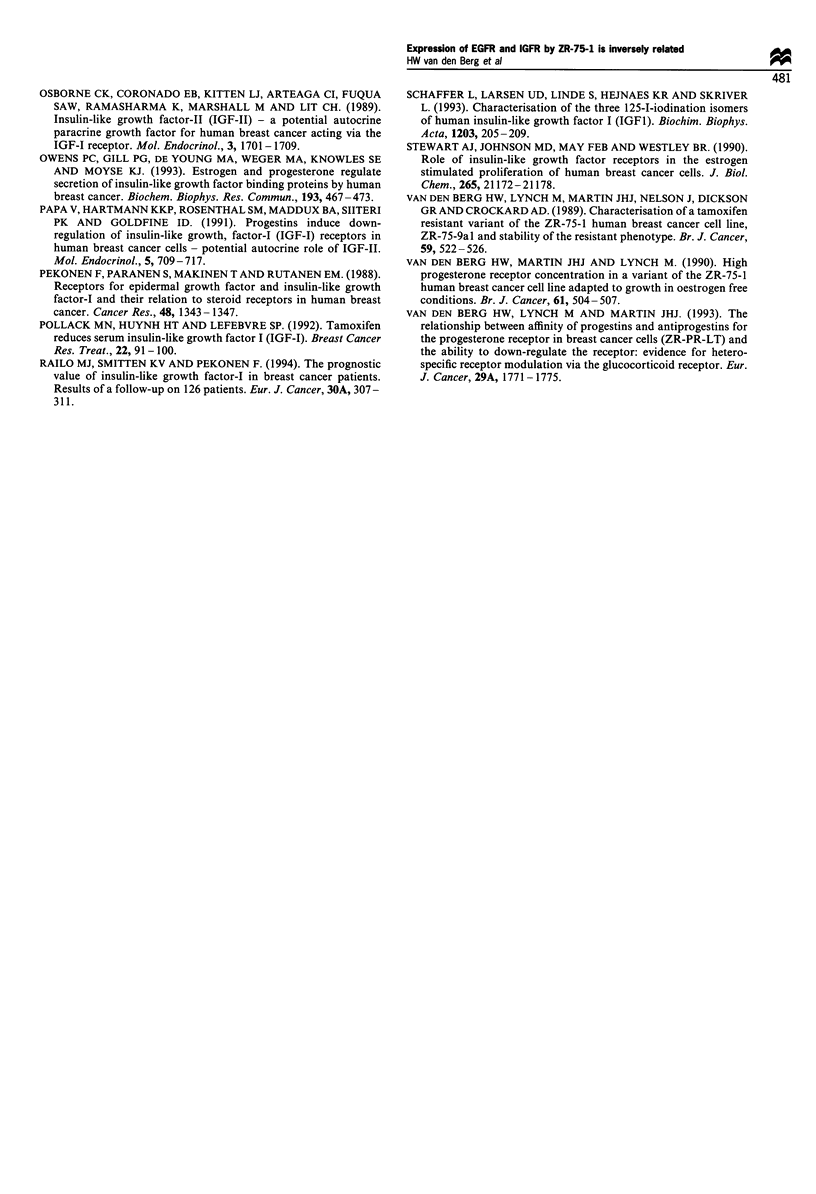


## References

[OCR_00581] Andreatta-Van Leyen S., Hembree J. R., Eckert R. L. (1994). Regulation of insulin-like growth factor 1 binding protein 3 levels by epidermal growth factor and retinoic acid in cervical epithelial cells.. J Cell Physiol.

[OCR_00588] Ewing T. M., Murphy L. J., Ng M. L., Pang G. Y., Lee C. S., Watts C. K., Sutherland R. L. (1989). Regulation of epidermal growth factor receptor by progestins and glucocorticoids in human breast cancer cell lines.. Int J Cancer.

[OCR_00593] Foekens J. A., Portengen H., van Putten W. L., Trapman A. M., Reubi J. C., Alexieva-Figusch J., Klijn J. G. (1989). Prognostic value of receptors for insulin-like growth factor 1, somatostatin, and epidermal growth factor in human breast cancer.. Cancer Res.

[OCR_00598] Fraker P. J., Speck J. C. (1978). Protein and cell membrane iodinations with a sparingly soluble chloroamide, 1,3,4,6-tetrachloro-3a,6a-diphrenylglycoluril.. Biochem Biophys Res Commun.

[OCR_00606] Hana V., Murphy L. J. (1994). Interdependence of epidermal growth factor and insulin-like growth factor-I expression in the mouse uterus.. Endocrinology.

[OCR_00611] Harris A. L., Nicholson S., Sainsbury J. R., Farndon J., Wright C. (1989). Epidermal growth factor receptors in breast cancer: association with early relapse and death, poor response to hormones and interactions with neu.. J Steroid Biochem.

[OCR_00616] Hembree J. R., Agarwal C., Eckert R. L. (1994). Epidermal growth factor suppresses insulin-like growth factor binding protein 3 levels in human papillomavirus type 16-immortalized cervical epithelial cells and thereby potentiates the effects of insulin-like growth factor 1.. Cancer Res.

[OCR_00623] Keightley D. D., Cressie N. A. (1980). The Woolf plot is more reliable than the Scatchard plot in analysing data from hormone receptor assays.. J Steroid Biochem.

[OCR_00628] Lahti E. I., Knip M., Laatikainen T. J. (1994). Plasma insulin-like growth factor I and its binding proteins 1 and 3 in postmenopausal patients with breast cancer receiving long term tamoxifen.. Cancer.

[OCR_00634] Long B., McKibben B. M., Lynch M., van den Berg H. W. (1992). Changes in epidermal growth factor receptor expression and response to ligand associated with acquired tamoxifen resistance or oestrogen independence in the ZR-75-1 human breast cancer cell line.. Br J Cancer.

[OCR_00641] Lønning P. E., Hall K., Aakvaag A., Lien E. A. (1992). Influence of tamoxifen on plasma levels of insulin-like growth factor I and insulin-like growth factor binding protein I in breast cancer patients.. Cancer Res.

[OCR_00644] Manni A., Badger B., Wei L., Zaenglein A., Grove R., Khin S., Heitjan D., Shimasaki S., Ling N. (1994). Hormonal regulation of insulin-like growth factor II and insulin-like growth factor binding protein expression by breast cancer cells in vivo: evidence for stromal epithelial interactions.. Cancer Res.

[OCR_00655] Nicholson S., Sainsbury J. R., Needham G. K., Chambers P., Farndon J. R., Harris A. L. (1988). Quantitative assays of epidermal growth factor receptor in human breast cancer: cut-off points of clinical relevance.. Int J Cancer.

[OCR_00665] Osborne C. K., Coronado E. B., Kitten L. J., Arteaga C. I., Fuqua S. A., Ramasharma K., Marshall M., Li C. H. (1989). Insulin-like growth factor-II (IGF-II): a potential autocrine/paracrine growth factor for human breast cancer acting via the IGF-I receptor.. Mol Endocrinol.

[OCR_00672] Owens P. C., Gill P. G., De Young N. J., Weger M. A., Knowles S. E., Moyse K. J. (1993). Estrogen and progesterone regulate secretion of insulin-like growth factor binding proteins by human breast cancer cells.. Biochem Biophys Res Commun.

[OCR_00677] Papa V., Hartmann K. K., Rosenthal S. M., Maddux B. A., Siiteri P. K., Goldfine I. D. (1991). Progestins induce down-regulation of insulin-like growth factor-I (IGF-I) receptors in human breast cancer cells: potential autocrine role of IGF-II.. Mol Endocrinol.

[OCR_00684] Pekonen F., Partanen S., Mäkinen T., Rutanen E. M. (1988). Receptors for epidermal growth factor and insulin-like growth factor I and their relation to steroid receptors in human breast cancer.. Cancer Res.

[OCR_00690] Pollak M. N., Huynh H. T., Lefebvre S. P. (1992). Tamoxifen reduces serum insulin-like growth factor I (IGF-I).. Breast Cancer Res Treat.

[OCR_00695] Railo M. J., von Smitten K., Pekonen F. (1994). The prognostic value of insulin-like growth factor-I in breast cancer patients. Results of a follow-up study on 126 patients.. Eur J Cancer.

[OCR_00701] Schäffer L., Larsen U. D., Linde S., Hejnaes K. R., Skriver L. (1993). Characterization of the three 125I-iodination isomers of human insulin-like growth factor I (IGF1).. Biochim Biophys Acta.

[OCR_00707] Stewart A. J., Johnson M. D., May F. E., Westley B. R. (1990). Role of insulin-like growth factors and the type I insulin-like growth factor receptor in the estrogen-stimulated proliferation of human breast cancer cells.. J Biol Chem.

[OCR_00724] van den Berg H. W., Lynch M., Martin J. H. (1993). The relationship between affinity of progestins and antiprogestins for the progesterone receptor in breast cancer cells (ZR-PR-LT) and ability to down-regulate the receptor: evidence for heterospecific receptor modulation via the glucocorticoid receptor.. Eur J Cancer.

[OCR_00714] van den Berg H. W., Lynch M., Martin J., Nelson J., Dickson G. R., Crockard A. D. (1989). Characterisation of a tamoxifen-resistant variant of the ZR-75-1 human breast cancer cell line (ZR-75-9a1) and ability of the resistant phenotype.. Br J Cancer.

[OCR_00718] van den Berg H. W., Martin J., Lynch M. (1990). High progesterone receptor concentration in a variant of the ZR-75-1 human breast cancer cell line adapted to growth in oestrogen free conditions.. Br J Cancer.

